# Intraamygdaloid Oxytocin Reduces Anxiety in the Valproate-Induced Autism Rat Model

**DOI:** 10.3390/biomedicines10020405

**Published:** 2022-02-08

**Authors:** Kristóf László, Orsolya Kiss, Dávid Vörös, Kitti Mintál, Tamás Ollmann, László Péczely, Anita Kovács, Olga Zagoracz, Erika Kertes, Veronika Kállai, Bettina László, Edina Hormay, Beáta Berta, Attila Tóth, Zoltán Karádi, László Lénárd

**Affiliations:** 1 Medical School, Institute of Physiology, University of Pécs, 7624 Pécs, Hungary; KPXBX4@pte.hu (O.K.); D7E59J@pte.hu (D.V.); kitti.mintal@aok.pte.hu (K.M.); tamas.ollmann@aok.pte.hu (T.O.); laszlo.peczely@aok.pte.hu (L.P.); anita.kovacs@aok.pte.hu (A.K.); olga.zagoracz@aok.pte.hu (O.Z.); erika.kertes@aok.pte.hu (E.K.); veronika.kallai@aok.pte.hu (V.K.); bettina.csetenyi@aok.pte.hu (B.L.); edina.hormay@aok.pte.hu (E.H.); beata.berta@aok.pte.hu (B.B.); attila.toth@aok.pte.hu (A.T.); zoltan.karadi@aok.pte.hu (Z.K.); laszlo.lenard@aok.pte.hu (L.L.); 2 Neuroscience Center, University of Pécs, 7624 Pécs, Hungary; 3 Szentágothai Center, Molecular Endocrinology and Neurophysiology Research Group, University of Pécs, 7624 Pécs, Hungary

**Keywords:** oxytocin, autism, valproate, amygdala, anxiety, rat, elevated plus maze

## Abstract

Background: Autism spectrum disorder (ASD) is a lifelong neurodevelopmental disorder affecting about 1.5% of children, and its prevalence is increasing. Anxiety is one of the most common comorbid signs of ASD. Despite the increasing prevalence, the pathophysiology of ASD is still poorly understood, and its proper treatment has not been defined yet. In order to develop new therapeutic approaches, the valproate- (VPA) induced rodent model of autism can be an appropriate tool. Oxytocin (OT), as a prosocial hormone, may ameliorate some symptoms of ASD. Methods: In the present study, we investigated the possible anxiolytic effect of intraamygdaloid OT on VPA-treated rats using the elevated plus maze test. Results: Our results show that male Wistar rats prenatally exposed to VPA spent significantly less time in the open arms of the elevated plus maze apparatus and performed significantly less head dips from the open arms. Bilateral OT microinjection into the central nucleus of the amygdala increased the time spent in the open arms and the number of head dips and reduced the anxiety to the healthy control level. An OT receptor antagonist blocked the anxiolytic effects of OT. The antagonist by itself did not influence the time rats spent in the open arms. Conclusions: Our results show that intraamygdaloid OT has anxiolytic effects in autistic rats.

## 1. Introduction

According to the 5th edition of the Diagnostic and Statistical Manual of Mental Disorders (DSM-5) [1], autism spectrum disorder (ASD) is characterized by permanent impairment of verbal and non-verbal social communication, interaction and existence of repetitive, restricted patterns of behavior, interest, or activities, and cognitive rigidity. Patients with ASD have difficulties in understanding other people’s emotion and in making eye contact. Delayed speech development and unexpected reactions to touch as well as to visual, auditory, gustatory, and olfactory stimuli are also characteristics of patients with ASD. ASD and intellectual disability also frequently co-occur. Mood disturbances are features of ASD too [1]. Patients with ASD may show extreme fear of harmless objects and/or a lack of fear of real danger [2]. Several types of anxiety can be associated with ASD, such as social anxiety disorder, generalized anxiety disorder, and separation anxiety disorder [2]. Murris et al. published that 84% of their ASD patients showed clinically significant anxiety [3]. In other studies, a lower rate (42–55%) of ASD patients met the criteria of clinically significant anxiety [4,5]. Despite the heterogenous prevalence, anxiety is considered one of the most common comorbid symptoms of ASD. Our study focused on the alleviation of anxiety in ASD patients.

Several animal models of autism (e.g., propionic acid-, intrauterine infection-, brain lesion-, thalidomide-, valproate (VPA)-induced models) have been developed in recent years to elucidate the detailed course of ASD. The VPA-induced model appears to be the most effective [6,7]. The three core characteristics of human autism, namely, social impairments, repetitive behavior, and cognitive rigidity, appear in VPA-treated rodents. Consequently, in the present study, the VPA-induced autism rat model was used in order to obtain rats with autistic-like behavior. VPA is a widely used, effective medication for epilepsy, bipolar disease, and migraine but it has a high teratogenic risk [8,9]. VPA increases the risk of neurodevelopmental failure in the fetus [10,11]. Several studies reported an increased risk of ASD in patients exposed to intrauterine VPA [11,12,13]. Based on the above data, it is reasonable that prenatal exposure of rodents to VPA provides a suitable model of ASD [6,7]. To obtain an autistic-like behavior in rodents, the dose and timing of VPA exposure are critical [6,7]. Kim et al. showed that the most significant changes in social behavior were obtained when rats were exposed to intrauterine VPA on the 12.5th day of gestation, but not on day 7, 9.5, and 15 [14]. As far as the VPA dose is concerned, 500 mg/bwkg of VPA evokes most effectively the autism-like symptoms in exposed offspring, without causing significant toxicological effects in dams [6,15]. Servadio et al. demonstrated that intrauterine VPA exposure (500 mg/bwkg i.p., gestational day 12.5) led to significantly decreased sociability, social play behavior, and pup distress calls [15]. They also showed that rats exposed to VPA (500 mg/bwkg i.p., gestational day 12.5) spent significantly less time in the open arms of the elevated plus maze [15]. Therefore, the anxiety level increased in rats showing autistic-like behavior [15].

In the present study, the behavioral effects of intraamygdaloid oxytocin (OT) were investigated using the elevated plus maze test and the VPA-induced rat model of ASD. Nonapeptide OT is a well-known pro-social hormone produced by supraoptic, paraventricular, and accessory nuclei of hypothalamus [16,17]. OT has been reported to play a role in pair bonding, parental care, social memory, sexual behavior, and affiliation [16,18].

The amygdala is an important part of the brain circuit regulating social behavior and anxiety [19]. The central nucleus of the amygdala (CeA) has been shown to receive OT fibers from the hypothalamus. Namely, the major sources of OT innervation are the accessory magnocellular nuclei, with additional oxytocinergic fibers from paraventricular and supraoptic nuclei [17]. It is also known that the CeA is rich in OT receptors [20,21].

Our previous studies showed that OT has positive reinforcing and anxiolytic effects when microinjected bilaterally into the CeA of intact Wistar rats [22]. Furthermore, our previous data also suggested that the activation of both the OT and the D2 dopamine (DA) receptors is necessary for the development of place preference and anxiolytic effects [23].

The main goal of the present study was to investigate the potential role of intraamygdaloid OT in anxiety in rats showing autistic-like behavior. The theoretical background of this goal is the following. Anxiety is one of the most common comorbid disorders found in ASD patients, and it has been clearly shown that anxiety appears in the VPA-induced rodent model of ASD [3,4,5,22]. The amygdala plays a key role in anxiety-related behavior, and amygdala hyperactivity was demonstrated in the VPA-induced autism model [24,25]. Furthermore, our previous findings showed anxiolytic effects of OT when microinjected into the CeA of healthy Wistar rats [22].

## 2. Materials and Methods

### 2.1. Subjects

The VPA-induced autism rat model was employed. Female rats in estrus phase (determined from vaginal smears) were mated overnight. Gestating female rats received a single intraperitoneal injection of 500 mg/bwkg valproate (Sigma-Aldrich Kft. Budapest, Hungary; P4543) dissolved in saline at a concentration of 250 mg/mL on the 12.5th day of gestation, and control females were injected with physiological saline. Dams were allowed to raise their own litters in their individual homecages. In our experiments, 42 male offspring Wistar rats weighing 270–290 g at the surgery were housed individually and cared for in accordance with institutional (BA02/2000-8/2012, BA02/2000-64/2017, BA02/2000-04/2021), national (Hungarian Government Decree, 40/2013 (II. 14.)), and international standards (European Community Council Directive, 86/609/EEC, 1986, 2010). The rats were kept in a temperature- and light-controlled room (22 ± 2 °C; 12:12 h light–dark cycle with lights on at 6:00 a.m.). Standard laboratory food pellets (CRLT/N standard rodent food pellet, Charles River Kft, Budapest, Hungary) and tap water were available ad libitum. The autistic like behavior of male offsprings was determined at the age of 4 and 8 weeks by means of social interaction test and open field test. We chose 42 (out of 67) VPA-exposed rats that showed less social interactions and more repetitive behavior than control rats. All behavioral tests were performed during the rats’ daylight period between 08:00 a.m. and 4:00 p.m.

### 2.2. Surgery

Rats were anesthetized i.p. by ketamine supplemented with diazepam (Calypsol and Seduxen, Richter Gedeon, Hungary, ketamine: 80 mg/kg body weight, diazepam: 20 mg/kg body weight). The animals were stereotaxically implanted bilaterally with 22-gauge stainless-steel guide cannulae, directed toward and 1 mm above the dorsal border of the CeA (coordinates relative to bregma: AP: −2.3 mm, ML: ±4.1 mm, DV: −6.5 mm) according to the rat stereotaxic atlas [26]. The cannulae were fixed to the skull with three stainless-steel screws and dental acrylic. When not used for injection, the guide cannulae were occluded with 27-gauge stainless-steel obturators. The animals were allowed a minimum of 7 days postoperative recovery before starting the experiments, during which period they were handled daily.

### 2.3. Drugs and Injection Procedure

OT, obtained from Sigma (Sigma–Aldrich Co., St. Louis, MO, USA, O6379), was bilaterally microinjected. The applied dose was 10 ng (9.93 pmol) in 0.4 μL/per side volume. OT was dissolved in 0.15 M sterile saline solution containing 0.01 M Na acetate and 0.01 M phosphate-buffered saline (PBS, pH 7.4). Control animals received this solution bilaterally as a vehicle in equal volume to that used for OT injections. The OT receptor antagonist L-2540 [Sigma–Aldrich Co., L-368-899, 20 ng (16.92 pmol)/0.4 μL)] was diluted in 0.15 M saline solution containing 0.01 M Na acetate and 0.01 M phosphate-buffered saline (PBS, pH 7.4). Altogether, five groups of animals were involved in the EPM experiment: a control group (healthy intact rats), VPA (intrauterine valproate-treated animals showing autistic-like behavior) and VPA + 10 ng OT groups (intrauterine valproate-treated animals showing autistic-like behavior and receiving a bilateral intraamygdaloid treatment with 10 ng OT), a VPA + ANT + OT group (intrauterine valproate-treated animals showing autistic-like behavior and receiving OT pretreated with the OT receptor antagonist), and a VPA + ANT group (intrauterine valproate-treated animals showing autistic-like behavior and receiving the OT receptor antagonist). The solutions were kept t + 4 °C before application. In this article, doses are reported as dose per side values. Drugs or vehicles were bilaterally microinjected through a 30-gauge stainless-steel injection tube extending 1 mm below the tips of the implanted guide cannulae. The injection cannula was attached via polyethylene tubing (PE-10) to a 10 μL Hamilton microsyringe (Hamilton Co., Bonaduz, Switzerland). All injections were delivered by a syringe pump in a 0.4 μL volume (Cole Parmer, Vernon Hills, IL, USA, IITC, Life Sci. Instruments, California) over a 60 s interval. After injection, the cannulae were left in place for additional 60 s to allow diffusion into the surrounding tissue. During the injections, the rats were gently held in hand. The test period started 5 min after the microinjections.

### 2.4. Elevated plus Maze Test (EPM)

Anxiety was evaluated in the EPM test. The apparatus was constructed using grey-colored wooden planks consisting of two opposite open arms (50 × 10 cm) and two opposite enclosed arms (50 × 10 × 40 cm), with an open roof. The maze was elevated to a height of 100 cm above the floor. After drug administration, the animals were placed into the center of the maze (central platform), facing one of the enclosed arms. The trials lasted 5 min, during which the number of entries into each arm, the time spent on the open and enclosed arms, and the number of head dips were recorded based on the protocol published in “The use of the elevated plus maze as an assay of anxiety-related behavior in rodents” [27]. Each rat was tested only once. The elevated plus maze apparatus was cleaned, deodorized with Quatricide, and dried with paper towels before testing the next rat [27]. Data were stored, and motion analysis was made by means of EthoVision Basic software (Noldus Information Technology B.V., Wageningen, The Netherlands).

### 2.5. Histology

At the end of the experiments, the rats received an overdose of Calypsol and Seduxen mixed in the ratio of 4:1 and were transcardially perfused with isotonic saline followed by a 10% formalin solution. After 1 week of from fixation, the brains were frozen, cut into 40 μm serial sections, and stained with Cresyl violet. The injection sites were reconstructed according to the stereotaxic atlas of the rat brain [26]. Only data from rats with correctly placed cannulae were analyzed.

### 2.6. Statistical Analysis

Data are presented as mean ± standard error of the mean (S.E.M.). One-way ANOVA followed by Tukey’s post hoc analysis and effect size estimation (eta squared for ANOVAs and Cohen’s d for pair-wise comparisons) were employed. Statistical significance was established at *p* < 0.05 (IBM SPSS Statistics 26).

## 3. Results

### 3.1. Histology

**Figure 1:**
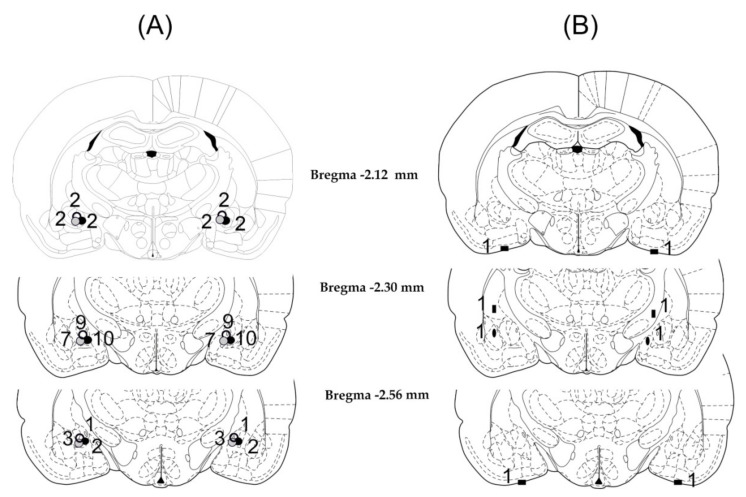
Illustration of reconstructed injection sites. Correct bilateral injection placements are indicated as circles in the CeA in panel (**A**) (*n* = 38). Incorrect injection placements are indicated in panel (**B**) (*n* = 4). Brain structure diagrams of coronal sections are adapted from the stereotaxic atlas of Paxinos and Watson. The numbers refer to the anterior–posterior distance from the bregma in mm. Identical symbols in panel B indicate coherent injection sites of bilateral injections. Numbers above marked sites in panel A and B indicate the number of animals.

Histological examination showed that in 38 of the 42 animals, the cannulae were precisely and symmetrically located in the target area (CeA). The tracks of the cannulae and the tip positions were determined by evidence of debris and moderate glial proliferation. A schematic illustration of cannulae placements is shown in Figure 1.

The four rats with misplaced injection sites were excluded from subsequent analysis (Figure 1B). Among these rats, in two cases, the cannula tips had symmetrically entered into the liquor space at the basis of the brain. In one case, the cannula tips were located laterally or medially and 1 mm above the AMY, thus the injections were made in the caudate putamen on one side and in the internal capsule on the other side. In one case, the cannula tips were placed laterally or medially to the target area; therefore, the injections were made in the lateral and basolateral AMY or in the medial AMY nucleus. Behavioral data concerning these incorrect and diverse placements were not enough to draw far-reaching conclusions.

### 3.2. Elevated plus Maze Test

**Figure 2:**
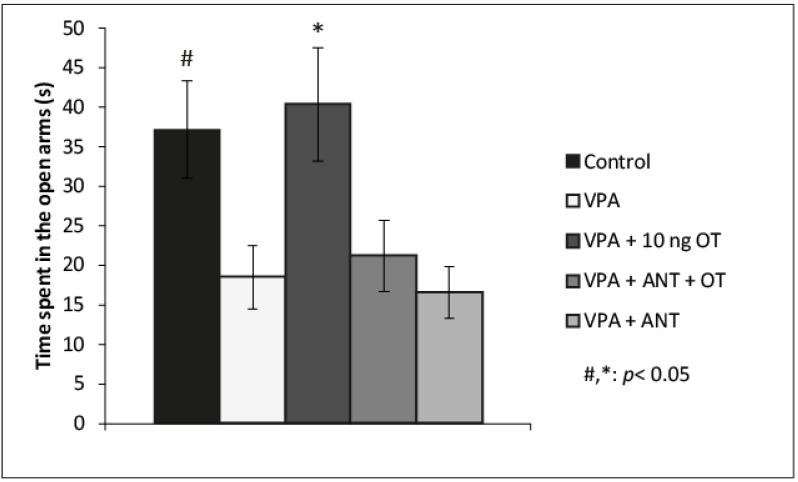
Effects of OT and the OT receptor antagonist injected in the CeA in autistic rats in the elevated plus maze (EPM) test. Columns represent the mean time (±S.E.M.) spent in the open arms. Control: healthy male Wistar rats receiving intraamygdaloid PBS (*n* = 8); VPA: intrauterine VPA-treated rats showing autistic-like behavior (*n* = 8); VPA + 10 ng OT: animals showing autistic-like behavior, microinjected with 10 ng OT (*n* = 8); VPA + ANT + OT: animals with autistic-like behavior, microinjected with 20 ng OT receptor antagonist and 10 ng OT (*n* = 7); VPA + ANT: animals with autistic-like behavior, microinjected with 20 ng OT receptor antagonist (*n* = 7); #, * *p* < 0.05, for more explanations, see the text.

The effects of VPA and intraamygdaloid OT and OT receptor antagonist on the time spent in the open arms during EPM are shown in Figure 2. Based on one-way ANOVA analysis, there was a significant effect among the groups [F(4, 33) = 4.387, η2 = 0.347, *p* < 0.01]. Tukey post hoc test revealed that VPA + 10 ng OT-treated rats (*n* = 8) spent significantly more time in the open arms compared to the VPA group (*n* = 8, *p* < 0.05, d = 1.326) or VPA + ANT + OT (*n* = 7, *p* < 0.05, d = 1.131) or the VPA + ANT-treated group (*n* = 7, *p* < 0.05, d = 1.488). There was no significant difference between control rats and VPA + 10 ng OT-treated rats (*n* = 8). OT receptor antagonist pretreatment prevented the anxiolytic effect of 10 ng OT. The OT receptor antagonist by itself did not influence the time spent in the open arms by VPA rats. There was no statistical difference among the VPA-, VPA + ANT + OT-, and VPA + ANT-treated group in relation to the time spent in the open arms. Tukey post hoc test also revealed that control rats (*n* = 8) spent significantly more time in the open arms compared to the VPA group (*n* = 8, *p* < 0.05, d = 3.588). To avoid the litter effect, only one rat was chosen from each litter of a given group.

**Figure 3:**
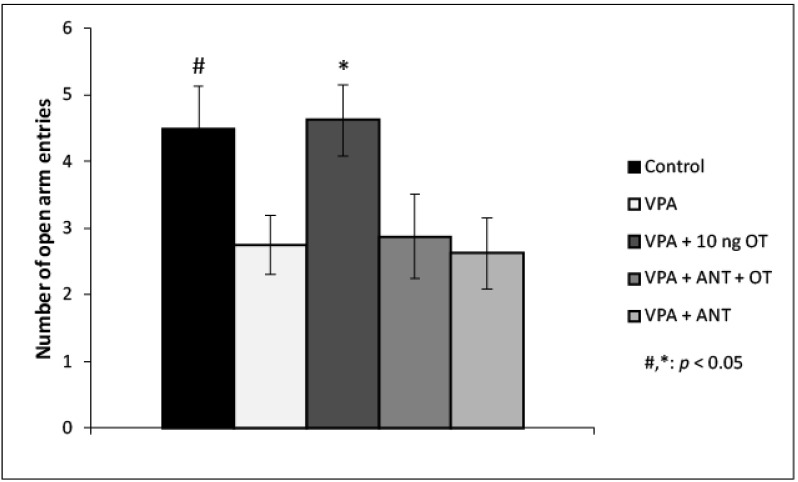
Effects of OT and OT receptor antagonist injected in the CeA in autistic rats in the elevated plus maze (EPM) test. Columns represent number of open arm entries (±S.E.M.). Control: healthy male Wistar rats receiving intraamygdaloid PBS (*n* = 8); VPA: intrauterine VPA-treated rats showing autistic-like behavior (*n* = 8); VPA + 10 ng OT: animals showing autistic-like behavior, microinjected with 10 ng OT (*n* = 8); VPA + ANT + OT: animals with autistic-like behavior, microinjected with 20 ng OT receptor antagonist and 10 ng OT (*n* = 7); VPA + ANT: animals with autistic-like behavior, microinjected with 20 ng OT receptor antagonist (*n* = 7); #, * *p* < 0.05, for more explanations, see the text.

The effects of VPA and intraamygdaloid OT and OT receptor antagonist on the number of open arm entries during EPM is shown in Figure 3. Based on one-way ANOVA analysis, there was a significant effect among the groups [F(4, 33) = 3.162, η2 = 0.265, *p* < 0.05]. Tukey post hoc test revealed that the VPA + 10 ng OT-treated rats (*n* = 8) entered the open arms significantly more times compared to the VPA group (*n* = 8, *p* < 0.05, d = 3.79), the VPA + ANT + OT- (*n* = 7, *p* < 0.05, d = 2.976), or the VPA + ANT-treated group (*n* = 7, *p* < 0.05, d = 3.757). There was no significant difference between control rats and VPA + 10 ng OT-treated rats (*n* = 8). OT receptor antagonist pretreatment prevented the anxiolytic effect of 10 ng OT. The OT receptor antagonist by itself did not influence the time spent in the open arms by the VPA rats. There was no statistical difference among the VPA-, VPA + ANT + OT-, and VPA + ANT-treated groups as far as the number of open arm entries was concerned. Tukey post hoc test also revealed that control rats (*n* = 8) entered the open arms significantly more times compared to the VPA group, (*n* = 8, *p* < 0.05, d = 3.19).

**Figure 4:**
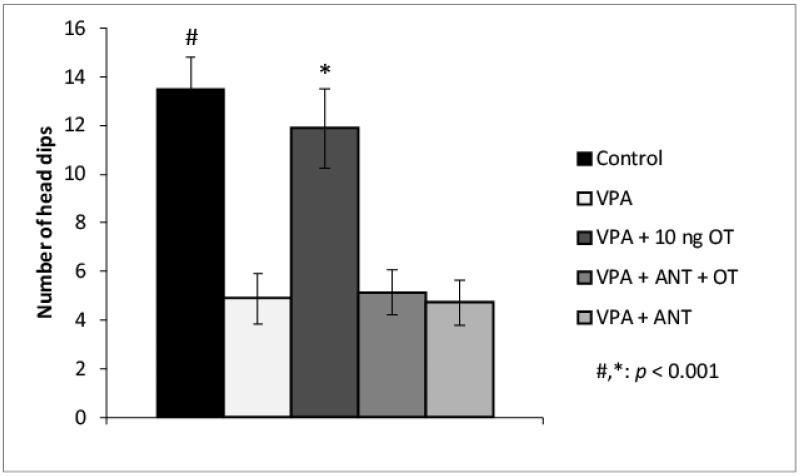
Effects of OT and OT receptor antagonist on the number of head dips in autistic rats in the elevated plus maze (EPM) test. Columns represent the mean number of head dips (±S.E.M.). Control: healthy male Wistar rats receiving intraamygdaloid PBS (n=8); VPA: intrauterine VPA-treated rats showing autistic-like behavior (*n* = 8); VPA + 10 ng OT: animals showing autistic-like behavior, microinjected with 10 ng OT (*n* = 8); VPA + ANT + OT: animals with autistic-like behavior, microinjected with 20 ng OT receptor antagonist and 10 ng OT (*n* = 7); VPA + ANT: animals with autistic-like behavior, microinjected with 20 ng OT receptor antagonist (*n* = 7); #, * *p* < 0.001, for more explanations, see the text.

The effects of intraamygdaloid OT and OT receptor antagonist head dipping in the EPM is shown in Figure 4. Based on one-way ANOVA analysis, there was a significant effect among the groups [F(4, 33) = 12.345, η2=0.599, *p* < 0.001]. Tukey post hoc test revealed that VPA + 10 ng OT-treated rats (*n* = 8) performed significantly more head dips compared to the VPA- (*n* = 8, *p* < 0.05, d = 1.8), the VPA + ANT +OT- (*n* = 7, *p* < 0.05, d = 1.762), or the VPA + ANT-treated groups (*n* = 7, *p* < 0.05, d = 1.881). OT receptor antagonist pretreatment prevented the increase in the number of head dips. The OT receptor antagonist by itself did not influence the number of head dips in VPA rats. There was no statistical difference among the VPA-, VPA + ANT + OT-, and VPA + ANT-treated groups in regard to the number of head dips. Tukey post hoc test has also revealed that control rats (*n* = 8) showed significantly more head dips compared to the VPA group (*n* = 8, *p* < 0.05, d = 7.029).

Based on one-way ANOVA analysis, a very strong tendency was seen regarding the “% of open arm entry”, but it failed to reach a significant level [F(4, 35) = 2.445, *p* = 0.065] (Table 1). Control rats entered the open arms in 26.85 ± 3.02% of their total entries, whereas VPA rats entered the open arms only in 17.28 ± 2.92% of their total entries, and the chance that autistic rats, which received 10 ng OT, chose the open arms was 27.06 ± 2.56% (see Table 1 for more details). The sum of the entries into all arms was also measured, but there was no significant difference among the groups [F(4, 35) = 0.701, *p* = 0.597] (Table 1). Our data showed that there was no significant difference among the groups in regard to the covered distance [F(4, 35) = 0.821, *p* = 0.521] (Table 1).

## 4. Discussion

OT has shown a significant impact on empathy, trust, and social cognition [28,29]. Some studies indicated that the plasma OT level is significantly lower in patients with ASD [30]. Furthermore, correlations have been shown between OT levels and the severity of ASD [31]. A meta-analysis revealed that the OT receptor gene is associated with ASD [32]. It has been also shown that exogenous and evoked OT restore social behavior in the Cntnap2 mouse model of autism [33]. Moreover, an intranasal OT treatment was shown to improve the autistic-like behavior in mice in the open field test, the marble burying test, the tail suspension test, and the three-chamber social interaction test [34]. Intranasal OT treatment has been reported to alleviate autistic cognitive and mood dysfunctions in the VPA-induced rodent model of autism [35]. The aforementioned study found that OT improved the oxidative status and ameliorated short-term memory and depressive manifestations [35]. Evidence was found that central administration of OT was more effective than peripheral administration [35]. In humans, OT has been also demonstrated to promote social behavior in high-functioning ASD patients [36]. Despite a large number of preclinical and clinical trials, there is no effective therapy for ASD, so its treatment is still a challenge [37].

The VPA-induced autism rodent model is widely accepted [6]. It has been reported that VPA induces anxiety in rodents [24], and adolescent VPA rats exhibit a lower level of OT mRNA and fewer OT-immunoreactive (OT-ir) cells in the hypothalamus than control rats [38]. Additionally, OT concentration in the cerebrospinal fluid (CSF) is reduced in autistic rats compared to healthy controls [38]. It has been also demonstrated that the number of OT-ir cells in the supraoptic nucleus (SON) of neonatal VPA rats is lower [38]. Dai et al. have also shown that early postnatal OT treatment had a long-term therapeutic effect on autistic-like behaviors in VPA rats [38]. OT dysfunction can be observed not only in the VPA-induced autism model but also in the monogenic and multigenic heritable forms of autism [38]. The aforementioned results clearly suggest a role of the OT system in rodent models of autism, especially in the VPA-induced autism model.

OT receptors are widely distributed in the brain. It has been suggested that within the rat CeA (lateral division), 18.6 ± 1.8% of astrocytes and 67.8 ± 3.1% of neurons expressed the OT receptor mRNA [39]. OT receptor knockout mice were demonstrated to have impaired social cognition, perform less social interaction, and show other autistic-like behaviors [40,41]. The above data further support the important role of the OT system in ASD.

Anxiety is one of the most important comorbid diseases in ASD patients [1,3,4]. Anxiety can be also responsible for poorer outcomes of academic performance and social skills in autistic children [42]. It was also reported that anxiety can alter verbal fluency and may provoke repetitive and restrictive behaviors [43,44,45]. Consequently, anxiety may influence the severity of ASD and vice versa, thus predicting a poorer quality of life [43,44,45]. Furthermore, ASD children with anxiety have a greater risk to develop self-dangerous, disruptive behaviors and depressive symptoms [46]. In line with our results, other studies also indicated the anxiogenic effects of prenatal VPA treatment, resulting in autistic-like behavior [6,15]. Increased reactivity of the amygdala was also shown in the VPA-induced autism model [24]. One may suggest how intraamygdaloid OT is involved. OT has been reported to be a modulator of anxiety in the amygdala [17,20,22,23]. Our previous findings indicated that 10 ng OT had anxiolytic effects when microinjected into the CeA of intact male rats [22]. The novelty of the present study is that bilateral 10 ng OT microinjections into the CeA of VPA rats with autistic-like symptoms resulted in anxiolytic behavior. Namely, our results showed that the time spent by the VPA-exposed rats in the open arms of the elevated plus maze and the number of open arm entries increased following intraamygdaloid OT treatment and reached the healthy control level. One may suppose the anxiolytic effect of OT on the basis of its general inhibitory effect on amygdala activity [47].

The number of head dips was also evaluated in our experiments. Head dips are downward movements of rats’ head toward the floor in the open arms [27]. Anxiety behavior is associated with a decreased number of head dips [27]. Our results indicated that VPA rats showed significantly less head dips. The above-mentioned findings were predictable, since studies indicated an increased level of anxiety in rodents treated prenatally with VPA [6]. However, the novelty of our research is that intraamygdaloid OT treatment could increase the number of head dips in VPA-exposed rats. This effect was OT receptor-specific, since OT receptor antagonist pretreatment could block the effect of OT. Therefore, OT not only increased the time spent in the open arms by autistic rodents and the number of open arm entries but also increased the number of head dips, suggesting an anxiolytic effect. Male rats were tested in our experiments. Therefore, our data do not provide information on gender differences. It is important to emphasize that only one rat from one litter was assigned to each group. Consequently, the litter effect could be excluded.

Nowadays, the therapy of anxiety in ASD includes cognitive-behavioral therapy and psychopharmacological treatment [48]. Selective serotonin reuptake inhibitors [49] and serotonin–norepinephrine reuptake inhibitors are considered to be the first choice as pharmacological treatment of anxiety disorder in the general population. However, there is some evidence suggesting that individuals with ASD are vulnerable to the side effects of SSRIs, including insomnia, impulsivity, and hyperactivity [48]. OT treatment might have promising therapeutical potential for ASD, but further studies are required [50]. Our data may provide a basis for the determination of potential drug targets in therapeutic approaches and for other future preclinical–clinical research projects

## Figures and Tables

**Table 1:** Sum of entries into all arms (±S.E.M.), % of open arm entries (±S.E.M.), and covered distance (cm) (±S.E.M.) are shown in Table 1. Control: healthy male Wistar rats receiving intraamygdaloid PBS (*n* = 8); VPA: intrauterine VPA treated rats showing autistic-like behavior (*n* = 8); VPA + 10 ng OT: animals showing autistic-like behavior, microinjected with 10 ng OT (*n* = 8); VPA + ANT + OT: animals with autistic-like behavior, microinjected with 20 ng OT receptor antagonist and 10 ng OT (*n* = 7); VPA + ANT: animals with autistic-like behavior, microinjected with 20 ng OT receptor antagonist (*n* = 7).

	Sum of Entries	% of Open Arm Entry	Covered Distance (cm)
Control	16.62 ± 0.77	26.85 ± 3.02%	1852.00 ± 50.29
VPA	16.12 ± 0.51	17.28 ± 2.92%	1821.13 ± 54.66
VPA+10 ng OT	17.00 ± 0.73	27.06 ± 2.56%	1709.52 ± 66.72
VPA+ANT+OT	15.51 ± 0.92	18.44 ± 4.10%	1802.75 ± 67.89
VPA+ANT	15.37 ± 1.12	16.68 ± 3.41%	1728.51 ± 90.68

## References

[1] American Psychiatric Association (2013). Diagnostic and Statistical Manual of Mental Disorders.

[2] Van Steensel F.J., Bogels S.M., Perrin S. (2011). Anxiety Disorders in Children and Adolescents with Autistic Spectrum Disorders: A Meta-analysis. Clin. Child Fam. Psychol. Rev..

[3] Muris P., Steerneman P., Merckelbach H., Holdrinet I., Meesters C. (1998). Comorbid Anxiety Symptoms in Children with Pervasive Developmental Disorders. J. Anxiety Disord..

[4] De Bruin E.I., Ferdinand R. F., Meester S., de Nijs P. F. (2007). High Rates of Psychiatric Co-Morbidity in PDD-NOS. J. Autism Dev. Disord..

[5] Simonoff E., Pickles A., Charman T., Chandler S., Loucas T., Baird G. (2008). Psychiatric Disorders in Children with Autism Spectrum Disorders: Prevalence, Comorbidity, and Associated Factors in a Population-Derived Sample. J. Am. Acad. Child Adolesc. Psychiatry.

[6] Tartaglionea A.M., Schiavi S., Calamandreia G., Trezza V. (2018). Prenatal Valproate in Rodents as a Tool to Understand the Neuralunderpinnings of Social Dysfunctions in Autism Spectrum Disorder. Neuropharmacology.

[7] Mabunga D.F., Gonzales E.L.T., Kim J.-W., Kim K.C., Shin C.Y. (2015). Exploring the Validity of Valproic Acid Animal Model of Autism. Exp. Neurobiol..

[8] Ornoy A. (2006). Neuroteratogens in Man: An Overview with Special Emphasis on the Teratogenicity of Antiepileptic Drugs in Pregnancy. Reprod. Toxicol..

[9] Wieck A., Jones S. (2018). Dangers of Valproate in Pregnancy. BMJ Br. Med. J..

[10] Barrett C., Richens A. (2003). Epilepsy and Pregnancy: Report of an Epilepsy Research Foundation Workshop. Epilepsy Res..

[11] Bromley R.L., Mawer G.E., Briggs M., Cheyne C., Clayton-Smith J., García-Fiñana M., Kneen R., Lucas S.B., Shallcross R., Baker G.A. (2013). The Prevalence of Neurodevelopmental Disorders in Children Prenatally Exposed to Antiepileptic Drugs. J. Neurol. Neurosurg. Psychiatry.

[12] Christensen J., Grønborg T.K., Sørensen M.J., Schendel D., Parner E.T., Pedersen L.H., Vestergaard M. (2013). Prenatal Valproate Exposure and Risk of Autism Spectrum Disorders and Childhood Autism Editorial Comment. Obstet. Gynecol. Surv..

[13] Wood A.G., Nadebaum C., Anderson V., Reutens D., Barton S., O’Brien T., Vajda F. (2015). Prospective Assessment of Autism Traits in Children Exposed to Antiepileptic Drugs during Pregnancy. Epilepsia.

[14] Kim K.C. (2011). The Critical Period of Valproate Exposure to Induce Autistic Symptoms in Sprague-Dawley Rats. Toxicol. Lett..

[15] Servadio M., Manduca A., Melancia F., Leboffe L., Schiavi S., Campolongo P., Palmery M., Ascenzi P., di Masi A., Trezza V. (2018). Impaired Repair of DNA Damage Is Associated with Autistic-Like Traits in Rats Prenatally Exposed to Valproic Acid. Eur. Neuropsychopharmacol..

[16] Grinevich V., Knobloch-Bollmann H.S., Eliava M., Busnelli M., Chini B. (2016). Assembling the Puzzle: Pathways of Oxytocin Signaling in the Brain. Biol. Psychiatry.

[17] Knobloch H.S. (2012). Evoked Axonal Oxytocin Release in the Central Amygdala Attenuates Fear Response. Neuron.

[18] Lee H.J. (2009). Oxytocin: The Great Facilitator of Life. Prog. Neurobiol..

[19] DeMayo M.M. (2019). Circuits for Social Learning: A Unified Model and Application to Autism Spectrum Disorder. Neurosci. Biobehav. Rev..

[20] Bale T.L., Davis A.M., Auger A.P., Dorsa D.M., McCarthy M.M. (2001). CNS Region-Specific Oxytocin Receptor Expression: Importance in Regulation of Anxiety and Sex Behavior. J. Neurosci..

[21] Gimpl G., Fahrenholz F. (2001). The Oxytocin Receptor System: Structure, Function, and Regulation. Physiol. Rev..

[22] Laszlo K. (2016). Positive Reinforcing Effect of Oxytocin Microinjection in the Rat Central Nucleus of Amygdala. Behav. Brain Res..

[23] Laszlo K. (2020). The Role of D2 Dopamine Receptors in Oxytocin Induced Place Preference and Anxiolytic Effect. Horm. Behav..

[24] Markram K., Rinaldi T., La Mendola D., Sandi C., Markram H. (2008). Abnormal Fear Conditioning and Amygdala Processing in an Animal Model of Autism. Neuropsychopharmacology.

[25] Thomason M., Austin A., Hendrix C. (2021). Fetal Amygdala Functional Connectivity Relates to Autism Spectrum Disorder Traits at Age 3. Biol. Psychiatry.

[26] Paxinos G., Watson C. (1986). The Rat Brain in Stereotaxic Coordinates.

[27] Walf A.A., Frye C.A. (2007). The Use of the Elevated Plus Maze as an Assay of Anxiety-Related Behavior in Rodents. Nat. Protoc..

[28] Guastella A.J., MacLeod C. (2012). A Critical Review of the Influence of Oxytocin Nasal Spray on Social Cognition in Humans: Evidence and Future Directions. Horm. Behav..

[29] Kosfeld M. (2005). Oxytocin Increases Trust in Humans. Nature.

[30] Modahl C. (1998). Plasma Oxytocin Levels in Autistic Children. Biol. Psychiatry.

[31] Hammock E., Veenstra-VanderWeele J. (2012). Examining Autism Spectrum Disorders by Biomarkers: Example from the Oxytocin and Serotonin Systems. J. Am. Acad. Child Adolesc. Psychiatry.

[32] LoParo D., Waldman I.D. (2015). The Oxytocin Receptor Gene (OXTR) Is Associated with Autism Spectrum Disorder: A Meta-Analysis. Mol. Psychiatry.

[33] Penagarikano O. (2015). Exogenous and Evoked Oxytocin Restores Social Behavior in the Cntnap2 Mouse Model of Autism. Sci. Transl. Med..

[34] Wang Y. (2018). Oxytocin Improves Animal Behaviors and Ameliorates Oxidative Stress and Inflammation in Autistic Mice. Biomed. Pharmacother..

[35] Lefter R. (2020). Oxytocin Differentiated Effects According to the Administration Route in a Prenatal Valproic Acid-Induced Rat Model of Autism. Med. Lith..

[36] Andari E. (2010). Promoting Social Behavior with Oxytocin in High-Functioning Autism Spectrum Disorders. Proc. Natl. Acad. Sci. USA.

[37] Huang Y. (2021). Intranasal Oxytocin in the Treatment of Autism Spectrum Disorders: A Multilevel Meta-Analysis. Neurosci. Biobehav. Rev..

[38] Dai Y.C. (2018). Neonatal Oxytocin Treatment Ameliorates Autistic-Like Behaviors and Oxytocin Deficiency in Valproic Acid-Induced Rat Model of Autism. Front. Cell. Neurosci..

[39] Wahis J. (2021). Astrocytes Mediate the Effect of Oxytocin in the Central Amygdala on Neuronal Activity and Affective States in Rodents. Nat. Neurosci..

[40] Zhang R. (2017). The Role of the Oxytocin/Arginine Vasopressin System in Animal Models of Autism Spectrum Disorder. Adv. Anat. Embryol. Cell. Biol..

[41] Pobbe R.L. (2012). Oxytocin Receptor Knockout Mice Display Deficits in the Expression of Autism-Related Behaviors. Horm. Behav..

[42] Ambrose K., Adams D. (2020). Exploring Profiles of Anxiety Symptoms in Male and Female Children on the Autism Spectrum. Res. Autism Spectr. Disord..

[43] Kerns C.M. (2016). The Treatment of Anxiety in Autism Spectrum Disorder (TAASD) Study: Rationale, Design and Methods. J. Child Fam. Stud..

[44] Kimura Y. (2020). An Investigation of the Effect of Social Reciprocity, Social Anxiety, and Letter Fluency on Communicative Behaviors in Adults with Autism Spectrum Disorder. Psychiatry Res..

[45] Wood J.J., Gadow K.D. (2010). Exploring the Nature and Function of Anxiety in Youth with Autism Spectrum Disorders. Clin. Psychol. Sci. Pract..

[46] Kerns C.M. (2015). Not to Be Overshadowed or Overlooked: Functional Impairments Associated with Comorbid Anxiety Disorders in Youth with ASD. Behav. Ther..

[47] Sobota R. (2015). Oxytocin Reduces Amygdala Activity, Increases Social Interactions, and Reduces Anxiety-Like Behavior Irrespective of NMDAR Antagonism. Behav. Neurosci..

[48] Postorino V. (2017). Anxiety Disorders and Obsessive-Compulsive Disorder in Individuals with Autism Spectrum Disorder. Curr. Psychiatry Rep..

[49] Kaskous S.H. (2006). Oxytocin Release and Lactation Performance in Syrian Shami Cattle Milked with and without Suckling. J. Dairy Res..

[50] Bernaerts S. (2020). Behavioral Effects of Multiple-Dose Oxytocin Treatment in Autism: A Randomized, Placebo-Controlled Trial with Long-Term Follow-up. Mol. Autism.

